# Healthcare utilization and economic burden of myopia in urban China: A nationwide cost-of-illness study

**DOI:** 10.7189/jogh.12.11003

**Published:** 2022-03-19

**Authors:** Yingyan Ma, Yuechun Wen, Hua Zhong, Senlin Lin, Li Liang, Yifang Yang, Huifen Jiang, Jian Chen, Yan Huang, Xiaohua Ying, Serge Resnikoff, Lina Lu, Jianfeng Zhu, Xun Xu, Xiangui He, Haidong Zou

**Affiliations:** 1 Department of Preventative Ophthalmology, Shanghai Eye Disease Prevention and Treatment Center, Shanghai Eye Hospital, Shanghai, China; 2 Department of Ophthalmology, First Affiliation Hospital of University of Science and Technology of China, Anhui, China; 3 Department of Ophthalmology, First Affiliated Hospital of Kunming Medical University, Yunnan, China; 4 Department of Ophthalmology, Shanghai General Hospital, School of Medicine, Shanghai Jiao Tong University, Shanghai, China; 5 Baoshan District Center for Disease Control and Prevention, Shanghai, China; 6 Huangpu Center for Disease Control and Prevention, Shanghai, China; 7 Shanghai Putuo District Dental Clinic & Department of Ophthalmology Clinic, Shanghai, China; 8 Department of Health Economics, School of Public Health, Fudan University, Shanghai, China; 9 Brien Holden Vision Institute, Sydney, Australia; 10 School of Optometry and Vision Science, University of New South Wales, Sydney, Australia

## Abstract

**Background:**

China contributes to a significant proportion of the myopia in the world. The study aims to investigate the utilization of various correction methods and health service in urban China, and to estimate the cost of myopia treatment and prevention. In addition, we aimed to estimate the cost of productivity loss due to myopia.

**Methods:**

The study was a cross-sectional investigation carried out in urban areas in three provinces located in the east (Shanghai), middle (Anhui) and west part (Yunnan) of China, in 2016. A total of 23819 people aged between 5 to 50 years were included. Health utilization and the cost of myopia were analyzed from patients’ perspective.

**Results:**

The total number of people with myopia in the urban China was estimated to be 143.6 million. The correction rate was 89.5%, 92.1%, and 92.7% for Anhui, Shanghai, and Yunnan (χ^2^ = 19.5, *P* < 0.01). Over the recent year, 20.6%, 16.8%, and 28.8% of myopic subjects visited hospital due to myopia, in Anhui, Shanghai and Yunnan. The annual cost of treatment and prevention of myopia was 10.1 billion US dollar (US$, floating from 9.2 to 11.2 billion US$), and the cost per person was 69US$. The annual cost of loss of productivity was estimated to be 6.7 billion US$ for those with mild to moderate visual impairment (floating from 6.1 to 7.4 billion US$), and 9.4 billion US$ (floating from 8.5 to 10.4 billion US$) for those with severe visual impairment to blindness. Therefore, the total economic burden of myopia was estimated as 173.6 billion CNY (26.3 billion US$).

**Conclusions:**

The present study shows that myopia leads to substantial economic burden in China. The loss of productivity caused by myopia is an important part of the disease burden compared to the cost of correction and treatment paid by individuals. Therefore, the focus of myopia prevention and control should be to decrease the myopia prevalence, and prevent the uncorrected refractive errors and the irreversible damage of visual acuity by high myopia.

Myopia has become an important public health issue. In 2010 there were 1950 million (prevalence of 28.3%) myopic people worldwide, and it is estimated that this number will increase to 4758 million (prevalence of 49.8%) in 2050 [1]. The costs of correcting myopia, treating the progression of the disease and its complications could bring heavy economic burden to both individuals and society. Meanwhile, myopia accounts for the major proportion of uncorrected refractive error, which is one of the leading causes of blindness and low vision [2]. Additionally, complications resulting from myopia and high myopia, such as glaucoma, retinopathy, and retinal detachment, are also major causes of blindness and low vision [3]. It was estimated that the global potential productivity loss associated with the burden of myopia related visual impairment in 2015 was about 250 billion US dollar (US$) [4].

The utilization of correction methods, health service, and the composition of the cost in China have been unidentified. Spectacles, contact lenses and surgery, are the most common tools for correcting the myopia; and their cost varies widely. The use of orthokeratology or rigid gas permeable (RGP) lenses has shown to be effective in slowing the progression of myopia [5,6]; nevertheless, with the expensive costs and frequent follow-up, the utilization rate of this tool remains undefined. Compared with soft contact lenses, laser surgery has proven to be cost-effective in correcting myopia in adults in developed countries [7,8]; yet, the surgery rate in the myopic population in developing countries such as in China, remains undefined. For myopic children, the refraction measurement usually requires cycloplegia; however, some patients chose to use spectacle stall instead of going to hospital, thus not fully utilizing the medical health service. Moreover, prevalence of myopia is especially high in young Chinese children [9], who need to be accompanied when visiting the clinic or spectacle stalls. The exact indirect costs associated with accompany by parents or relative compared with direct costs remains undefined. Identifying all these issues related to the burden of myopia could help decision makers to judge the rationality of the utilization and the allocation of the resources and to formulate policies that would improve the current situation, thus relieving the burden of myopia.

In addition, there are limited reports on the economic burden of myopia. In Singapore, calculation has indicated that the approximate annual cost for treating myopia is 755 million US$ [10,11]. In the United States, the annual direct cost of correcting distance vision caused by refractive errors including myopia goes from 3.8 billion to 7.2 billion US$ [12]. In developed countries, the economic burden of myopia is relatively heavy no matter the prevalence of myopia is high, eg, in Singapore, or low, eg, United States. In developing countries with high prevalence of myopia, such as China, the economic burden remains unknown. In some urban areas of China, the prevalence of myopia is higher than 80% in the 18-year-olds [13]. Considering the large population of China, the economic burden caused by myopia could be relatively heavy; however, there is no literature on specific costs related to correcting or treating myopia or economic burden of myopia in China.

Based on the existing literature, we assume that myopia has caused a huge disease burden in China, and the indirect disease burden caused by myopia may account for a large part of the proportion. The present study aims to evaluate utilization and costs of various correction methods, health service in and outside the hospital, prevention measures and loss of productivity associated with myopia, and to calculate disease related annual cost in the urban China from the patient perspective.

## METHODS

### Study time and place

The study was carried out during November and December in 2016. According to the distribution of natural resources and the level of economic and social development of the provincial administrative regions, China is roughly divided into three economic zones: the east, the middle, and the west, representing the upper, medium and lower social economic status [14]. Considering possible influencing factors for disease economic burden, three provinces were randomly selected from the three economic zones. Shanghai in the east, Anhui in the middle and Yunnan in the west were included in the study.

### Study population

The study population were Chinese people aged 5 to 50 years in the urban areas of China, including myopic and non-myopic individuals. This population was chosen due to: 1) the prevalence of myopia in children younger than 5 years is relatively low (usually below 5% [9]); 2) people >50 years old are more commonly affected by presbyopia, which might confound the cost of spectacles for correcting myopia; 3) compared with people in the urban areas, people in the rural areas usually lack medical service and uncorrected or under corrected for refractive error [15]. In addition, the prevalence of myopia is much higher in the urban areas than in the rural areas in China [16].

### Sampling method, inclusion and exclusion criteria

In urban areas of each province, at least two primary schools, 2 middle schools, 2 high schools or schools of equivalent educational level, and two universities or schools of equivalent educational level were selected by random cluster sampling method. For children in the primary schools, middle schools and high schools, all children and their guardians were invited to participate in the investigation. Students and parents were included in the study, if their parents signed the written informed consent under the agreement with their children. For university students, all students were invited, and the students were included if they signed the written informed consent themselves.

### Sample size

Sample size was calculated according to prevalence of myopia at different age intervals, considering the cluster design effect of 2.0 and response rate of 85%, to attain 95% confidence intervals (Uα = 1.96) with a precision of 0.05 (δ). According to the formula N = Uα2*P*(1-P)/δ2, at least 760, 906, 760, and 760 people were required for primary school children (with an estimated myopia prevalence (P) of 30%), middle and high school children (50% prevalence), university students (70% prevalence) and the parents (30% prevalence), respectively, in each province.

### Questionnaire and its reliability

For primary school, middle school and high school students, the questionnaire was composed of three sections, ie, children, father and mother section. For university students, only one section was provided. The questionnaire consisted of 6 to 7 parts in each section. For students of primary school, middle school and high school, sections included myopia status, corrections methods, myopia treatment within the hospital, myopia treatment outside hospital, prevention and others. For university students and parents, additional sections including myopia surgery and family information were requested.

To verify the reliability of the questionnaire, before the study, we randomly selected two classes in a primary school and 2 classes in a middle school. A total of 96 students were included. The students and parents were asked to fill the questionnaire and repeated after 2 weeks. Total costs of myopia were calculated for each student and the parent, and the Pearson correlation coefficients were 0.98 (*P* < 0.01) and 0.96 (*P* < 0.01) for the student questionnaire and the parents questionnaire, respectively.

### Quality control

To guarantee the quality of completing the questionnaire, the investigators in each province were trained uniformly according to the self-written guide manual. Before filling the questionnaire, the investigators were required to fully explain the purpose and the methods of the study, and detailed information about how to fill the questionnaire. For university students, the investigators distributed the questionnaire and explained. The students filled the questionnaire on the spot; if they had any questions, they consulted the investigators. For students in the primary schools, middle schools and high schools and their parents, the questionnaire was filled by the parents. The investigators organized a parent’s meeting to explain the purpose of the study, and they established an online group to answer questions. If the parents were not able to participate, other guardians of the children were asked to fill the questionnaire.

The investigators randomly selected 10 questionnaires in every class to check the quality. If more than 10% of the items were missing or were not in accordance with logic or reality, the questionnaire was defined as unqualified and was returned to the students, which were kindly asked to fill the form once again. If more than two unqualified questionnaires were found in a class, another 10 were chosen and checked. The whole class was required to start over, if once again, more than 2 unqualified questionnaires were found.

Data from the questionnaire were entered into EpiData version 3.1 (The EpiData Association, Odense, Denmark), which could be checked automatically while entering the data. After entering the data, quality was assessed by checking the data entry variables against the original entries in the questionnaire. About 10% of the questionnaires was randomly selected for assessing the quality of data entry.

### Calculation of costs and sensitivity analyses

To avoid recall bias, if the spectacles and orthokeratology or RGP contact lenses were purchased more than 5 years ago, the cost was not included in the calculation for the cost per pair. The cost per pair was discounted if the spectacles or lenses were not purchased within the last year. Other items, such as frequency of replacement, and annual running cost were also calculated for all the people who response the questions. Annual running cost and annual cost for soft contact lenses were not discounted, since they were collected as cost generated during the most recent year. Utilization and cost for treating myopia or correcting myopia in institutions outside hospital, such as spectacles stores and private eye care institutions were also collected.

Costs for myopic laser surgery were calculated separately. The questionnaire items about myopic laser surgery were set for university students and parents, since in China, the surgery is only permitted for those aged 18 years and older. Those who underwent surgery within the recent 5 years were included to calculate the annual surgical rate and the average cost of surgery. The cost of surgery was discounted if it was not consumed in the most recent year. The annual cost of surgery was then calculated as the number of myopia multiple by the annual surgical rate and the average cost of surgery. Cost for prevention was calculated only in non-myopic children of primary school, middle school and high school.

Cost was calculated from patient’s perspective. We divided the total cost into two parts: 1) the cost of treatment and prevention of myopia, which consisted of both direct and indirect cost; and 2) the cost of loss of productivity caused by myopia, which only consisted of indirect cost.

To calculate the cost of treatment and prevention of myopia, the direct cost consisted of: 1) cost of correction, 2) medical cost created in hospital and transportation cost, 3) cost of myopia surgery and relevant medical cost, 4) cost created outside hospital for treating or correcting myopia and transportation cost, and 5) cost for preventing myopia, such buying eye-protection lamp, pencil grip, table and chair with sitting posture correction function. The indirect cost included: 1) cost of working time loss and cost of accompany by relatives because of myopia treatment in hospital and outside hospital, 2) cost of working time loss because of myopia surgery. Cost generated by working time loss was calculated as the hours off from work multiplied for the average wages per hour in 2016 in each province. Accompanying cost was calculated as the hours off from work multiplied for the average wages per hour in 2016 in each province and the number of accompanying people. In the baseline analysis, the cost was discounted for 3% to obtain the real value in 2016.

To evaluate the total annual cost of myopia treatment and prevention in the urban areas of China, we extrapolated the sample cost to the national wide population size. Using the sixth national census data of people aged 5 to 50 years in the urban China [17], and age specific prevalence of myopia reported in the present study, the amount of myopia and non-myopia people were obtained. To calculate the national-wide total cost, the number of people in each age interval was multiplied by the age specific utilization rate (eg, utilization rate of correction, medical health service, etc.), and multiplied for the median value of the corresponding cost. The total cost was analyzed using the following formula:



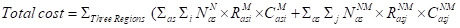



where *N_as_^M^* indicates age-group-specific regional myopia population, *R_asi_^M^* represents age-group-specific utilization rate of one health service for myopia treatment (eg, correction, out-patient and etc.), *C_asi_^M^* represents age-group-specific median value of one health service for myopia treatment (eg, correction, out-patient, etc.), *N_as_^NM^* stand for age-group-specific regional non-myopia population, *R_asj_^NM^* represents age-group-specific utilization rate of one health service for myopia prevention, and *C_asj_^NM^* indicates age-group-specific median value of one health service for myopia prevention.

To calculate the cost of loss of productivity caused by myopia, presenting distance visual acuity (PDVA) of the better eye collected by the questionnaire was extracted. The myopia subjects were defined as no visual impairment (PDVA≥ 0.6), mild visual impairment (PDVA≥ 0.3 and <0.6), moderate visual impairment (PDVA≥ 0.1, and <0.3), severe visual impairment (PDVA≥ 0.05, and <0.1), and blindness (PDVA<0.05). The proportions of the various types of visual impairment and blindness in myopia population were calculated for different provinces and age groups. Then, the number of people with various types of visual impairment and blindness were estimated from the number of people with myopia. Since the participants aged 5 to 20 years are mainly students and do not produce substantial social productivity, only those aged 20 to 50 years were included to estimate productivity loss. The productivity loss of patients with mild to moderate visual impairment is mainly caused by the decline in working ability. Therefore, we estimate their cost of productivity loss = number of patients with mild to moderate visual impairment × labor participation rate × per capita annual income loss_1_. However, the productivity loss of patients with severe visual impairment or blindness is mainly caused by the inability to participate in labor, so we estimate their cost of productivity loss = number of patients with severe visual impairment or blindness × labor withdrawal rate × per capita annual income loss_2_. We used the same labor participation/withdrawal rate as in the Professor Li's research, as well as the same per capita annual income loss_1_ and per capita annual income loss_2_ [18]. Sensitivity analyses were carried out by increasing and decreasing 1) the prevalence of myopia by 10%, 2) the per capita annual income loss_1_ and per capita annual income loss_2_ by 10%, and 3) floating discount rate from 0% to 5%. χ^2^ test and ANOVA test were used for comparing the differences of utilization rates and costs among the three provinces. Statistical analyses were performed using SAS 9.4 (SAS Institute, Inc, Cary, NC, USA). Costs are expressed in Chinese yuan (CNY) and US dollars (US$) using an exchange rate of US$ 1 = 6.6 CYN (as of June 1st 2016) [19].

### Patient and public involvement

All the participants were informed of the aim and potential benefit of the study for the public. The results of the present study will be sent to the government to formulate policies for myopia control in China.

### Ethics approval and consent to participate

The study was approved by the Shanghai General Hospital Ethics committee and adhered to the tenets of the Declaration of Helsinki. For students of primary school, middle school and high school, the parents signed the written informed consent under the agreement with their children. For university students, the students signed the written informed consent themselves.

## RESULTS

### Study population and prevalence of myopia

A total of 23 819 subjects, composed of 7206 subjects in Shanghai, 8430 in Anhui, and 8183 in Yunnan were invited. Subjects who did not sign the written informed consent, who were out of the study age span, or who didn’t fill the questionnaires effectively (who left more than 10% required filling items blank) were excluded from the analyses. Finally, a total of 22 097 (92.8%) subjects were included in the study. The basic characteristics of the study population were listed in **Table 1**.

**Table 1:** Basic characteristics of the study population *

	Anhui		Shanghai		Yunnan		Total		*P*-value†
**Age (years)**	**Male**	**Female**	**Male**	**Female**	**Male**	**Female**	**Male**	**Female**
5-9	399 (5.0)	347 (4.4)	362 (5.6)	343 (5.3)	282 (3.7)	324 (4.3)	1043 (4.8)	1014 (4.6)	0.04
10-14	535 (6.7)	461 (5.8)	391 (6.1)	347 (5.4)	489 (6.5)	521 (6.9)	1415 (6.5)	1329 (6.1)	0.04
15-19	562 (7.1)	563 (7.1)	429 (6.7)	459 (7.2)	355 (4.7)	609 (8.1)	1346 (6.1)	1631 (7.4)	<0.01
20-24	220 (2.8)	277 (3.5)	211 (3.3)	228 (3.6)	206 (2.7)	221 (2.9)	637 (2.9)	726 (3.3)	0.38
25-29	102 (1.3)	136 (1.7)	164 (2.6)	127 (2.0)	101 (1.3)	159 (2.1)	367 (1.7)	422 (1.9)	<0.01
30-34	295 (3.7)	482 (6.1)	163 (2.5)	261 (4.1)	227 (3)	471 (6.2)	685 (3.1)	1214 (5.5)	0.05
35-39	741 (9.3)	786 (9.9)	452 (7.0)	677 (10.6)	666 (8.8)	766 (10.3)	1859 (8.5)	2229 (10.2)	<0.01
40-44	783 (9.9)	691 (8.7)	550 (8.6)	573 (8.9)	847 (11.2)	672 (8.9)	2180 (1.0)	1936 (8.8)	<0.01
45-50	340 (4.3)	217 (2.7)	424 (6.6)	257 (4)	416 (5.5)	233 (3.1)	1180 (5.4)	707 (3.2)	0.54
Total	3977 (50.1)	3960 (49.9)	3146 (49.0)	3272 (51.0)	3589 (47.4)	3976 (52.6)	10 712 (48.9)	11 208 (51.1)	<0.01
Missing No.	26		23		128		177		

*The No. and the proportion (%) of the included people classified by age intervals and gender in each province were presented.

†χ^2^ test of gender composition of sample population among three provinces.

The prevalence of myopia calculated by self-reported questionnaire was listed in **Table 2**. All the three provinces showed a similar trend of myopia prevalence, which increased to over 80% in the 20-24 years of age, and then gradually decreased.

**Table 2:** Prevalence of myopia in the study population

	Anhui		Shanghai		Yunnan		All		*P*-value†
**Age (years)**	Myopia, No. (%)*	**Sample Size**	Myopia, No. (%)*	**Sample Size**	Myopia, No. (%)*	**Sample Size**	**Myopia, No. (%)**	**Sample Size**
5-9	81 (10.8)	750	130 (18.4)	706	30 (4.9)	607	241 (11.6)	2063	<0.01
10-14	361 (36.0)	1002	341 (45.7)	747	309 (30.5)	1012	1011 (36.6)	2761	<0.01
15-19	822 (72.6)	1133	637 (70.7)	901	620 (63.9)	971	2079 (69.2)	3005	<0.01
20-24	423 (83.8)	505	366 (83.4)	439	446 (82.0)	544	1235 (83.0)	1488	0.72
25-29	112 (47.1)	238	213 (73.2)	291	108 (41.4)	261	433 (54.8)	790	<0.01
30-34	187 (24.1)	777	184 (43.4)	424	111 (15.9)	698	482 (25.4)	1899	<0.01
35-39	339 (22.2)	1527	547 (48.5)	1129	266 (18.6)	1432	1152 (28.2)	4088	<0.01
40-44	329 (22.3)	1474	488 (43.5)	1123	294 (19.4)	1519	1111 (27.0)	4116	<0.01
45-50	115 (20.7)	557	269 (39.5)	681	149 (23.0)	649	533 (28.3)	1887	<0.01
All	2769 (34.8)	7963	3175 (49.3)	6441	2333 (30.3)	7693	8277 (37.5)	22 097	<0.01

*Myopia prevalence increased to over 80% in the 20-24 y of age, and then gradually decreased in all the three sampling areas.

†χ^2^ test of myopia prevalence of sample population among three provinces.

### Utilization of spectacles and contact lenses to correct myopia and the cost

**Figure 1:**
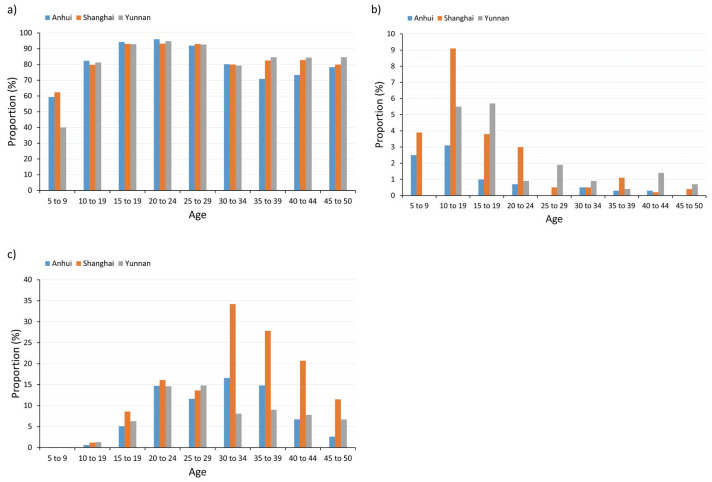
Spectacles and contact lenses utilization in three provinces. **Panel A**. Spectacles utilization rate in three provinces. **Panel B**. Orthokeratology or RGP utilization rate in three provinces. **Panel C**. Soft contact lenses utilization rate in three provinces.

Utilization of spectacles, orthokeratology or rigid gas permeable (RGP) lenses and soft contact lenses to correct myopia by age groups in the three provinces are shown in **Figure 1**. The percentage of person who reported to use any of the methods to correct myopia was 89.5%, 92.1%, and 92.7% in Anhui, Shanghai, and Yunnan, respectively (χ^2^ = 19.5, *P* < 0.01). The percentage was the lowest in children aged 5 to 9 years, being 69.1%, 71.5%, and 60% in Anhui, Shanghai, and Yunnan (χ^2^ = 1.53, *P* = 0.47). Using spectacles was the most popular way to correct myopia compared with the other two ways.

**Table 3** shows the calculation of the annual cost per person for spectacles, orthokeratology and RGP lenses, and soft contact lenses. The annual cost was the highest for the use of orthokeratology and RGP lenses, and the lowest for the use of spectacles. The mean of cost per pair of spectacles (F = 67.26, *P* < 0.01), frequency of replacement of spectacles (F = 19.72, *P* < 0.01), annual cost per person of spectacles (F = 34.53, *P* < 0.01), and annual cost per person of contact lenses (F = 8.8, *P* < 0.01) were significantly different among the three provinces.

**Table 3:** Costs and frequency of replacement of spectacles and contact lenses to correct myopia

Province		Variable	No.	Mean	SD	Median	Percentile
**Anhui**	Spectacles	Cost per pair, discounted (CNY)	1415	238.2	284.7	159.1	103.0-265.2
		Frequency of replacement (Year)	1968	2.1	1.6	2.0	1.0-3.0
		Annual cost per person (CNY)	1261	155.4	210.3	100.0	51.5-200.0
	OK and RGP lenses	Cost per pair, discounted (CNY)	11	4673.4	2546.3	4500.0	1500.0-6500.0
		Frequency of replacement (Year)	13	1.2	0.8	1.0	1.0-1.5
		Annual cost for lenses (CNY)	7	3107.1	1632.1	3000.0	1500.0-4500.0
		Annual running cost (CNY)	9	1726.7	1264.6	2100.0	480.0-2100.0
		Annual cost per person (CNY)	5	3762.0	2384.5	4500.0	1680.0-5100.0
	Soft contact lenses	Annual cost per person (CNY)	128	950.6	1166.7	480.0	180.0-1500.0
**Shanghai**	Spectacles	Cost per pair, discounted (CNY)	2135	555.4	1077.7	250.0	150.0-424.4
		Frequency of replacement (Year)	2330	2.4	1.8	2.0	1.0-3.0
		Annual cost per person (CNY)	1878	314.3	706.6	128.0	68.3-250.0
	OK and RGP lenses	Cost per pair, discounted (CNY)	57	5902.4	3511.0	5665.0	2575.0-8500.0
		Frequency of replacement (Year)	53	1.8	1.1	2.0	1.0-2.0
		Annual cost for lenses (CNY)	45	4189.3	4042.3	3250.0	1500.0-4500.0
		Annual running costs (CNY)	56	1610.4	1093.2	1500.0	720.0-2100.0
		Annual cost per person (CNY)	40	5822.1	4504.4	4870.0	2393.8-7700.0
	Soft contact lenses	Annual cost per person (CNY)	413	1740.2	2355.5	1500.0	480.0-2100.0
**Yunnan**	Spectacles	Cost per pair, discounted (CNY)	1627	470.9	690.6	273.2	200.0-412.0
		Frequency of replacement (Year)	1645	2.3	1.8	2.0	1.0-3.0
		Annual cost per person (CNY)	1308	303.0	550.8	152.3	77.3-300.0
	OK and RGP lenses	Cost per pair, discounted (CNY)	38	6465.6	2739.8	5665.0	4635.0-8500.0
		Frequency of replacement (Year)	39	1.6	0.9	2.0	1.0-2.0
		Annual cost for lenses (CNY)	30	4007.5	2344.7	3390.4	2317.5-5500.0
		Annual running costs (CNY)	47	1703.0	1194.2	1500.0	720.0-2700.0
		Annual cost per person (CNY)	27	5669.7	3059.5	6132.5	2853.3-8050.0
	Soft contact lenses	Annual cost per person (CNY)	156	1268.1	1462.6	1020.0	480.0-1500.0

OK – orthokeratology, RGP – rigid gas permeable, CNY – Chinese yuan, SD – standard deviation.

### Utilization of medical health service and the cost

Over the recent year, 20.6% (577/2796), 16.8% (553/3175), and 28.8% (673/2333) visited the hospital due to refraction measurement, contact lenses-induced infection, treatment with atropine and other treatment because of myopia, in Anhui, Shanghai and Yunnan province. In Anhui, the utilization rate was 64.2% for children aged 5 to 9 years, 44.6% for children aged 10 to 14 years, 23.8% for children aged 15 to 19 years, while it was 7.0%-13.7% for people of other age intervals. In Shanghai, the utilization rate was 81.5% for children aged 5 to 9 years, 55.4% for children aged 10 to 14 years, 16.5% for children aged 15 to 19 years, while it was 6.0%-9.8% for those of other age intervals. In Yunnan, the utilization rate was 73.3% for children aged 5 to 9 years, 66.7% for those aged 10 to 14 years, 37.4% for those aged 15 to 19 years, while it was 6.5%-20.1% for those in other age intervals.

Annual cost for using medical health services are listed in **Table 4**. Compared with the indirect cost, such as accompanying cost, the direct cost, including direct medical cost and traffic cost were relatively small in this section. The mean of direct medical cost (F = 7.22, *P* < 0.01), direct traffic cost (F = 8.11, *P* < 0.01), and cost of working time loss (F = 3.69, *P* = 0.03) were significantly different among the three provinces.

**Table 4:** Annual cost for using medical health service in hospital*

Province	Variable	No.	Mean (CNY)	SD	Median (CNY)	Percentile (CNY)
**Anhui**	Medical cost	577	109.5	246.1	4	0-80
	Traffic cost	577	37.1	181.1	0	0-15
	Cost of working time loss	541	38.0	138.2	0	0-25
	Cost of accompanying	505	263.2	1026.9	75	25-150
**Shanghai**	Medical cost	553	154.8	281.7	40	0-170
	Traffic cost	553	22.1	56.8	4	0-20
	Cost of working time loss	526	71.0	308.8	0	0-60
	Cost of accompanying	498	484.0	3245.5	180	60-360
**Yunnan**	Medical cost	673	170.2	325.9	20	0-180
	Traffic cost	673	59.5	203.3	2	0-50
	Cost of working time loss	638	89.1	430.4	0	0-30
	Cost of accompanying	590	512.5	1486.5	150	60-360

*Cost of working time loss were calculated as the hours off from work multiply the average wages per hour in 2016 in each province. For participants who underwent myopia surgery in the most recent year, the cost of using medical health service filled in this section was not calculated to avoid double calculation. For students of primary school, middle school and high school, cost of working time loss was not calculated. Cost of accompanying was calculated as the hours off from work multiply the average wages per hour in 2016 in each province and the number of people accompanying.

In Anhui, Shanghai and Yunnan, a total of 18, 17 and 16 myopic persons underwent myopic laser surgery in the most recent 5 years. The annual surgery rate was 0.19%, 0.15% and 0.21% in Anhui, Shanghai and Yunnan, respectively using the number of myopic persons in the university students and the parents’ population as the denominator.

The cost for myopic laser surgery was presented in **Table 5**. The medical cost included the cost for surgery, preoperative examinations, eye drops, follow-up and treating complications. No significant differences of the costs were found among the three provinces.

**Table 5:** Cost for myopic laser surgery*

Province	Variable	Mean (CNY)	SD	Median (CNY)	Percentile (CNY)
**Anhui**	Medical cost	9199.0	6392.6	6180	3376.5-15 000
	Cost of working time loss	1560.0	1962.8	600	400.0-2000
**Shanghai**	Medical cost	10 636.4	6225.3	10 000	6556.4-15913.5
	Cost of working time loss	4912.9	5565.5	3360	1440.0-3360
**Yunnan**	Medical cost	13 315.6	6734.9	11 255.09	7649.1-20 600
	Cost of working time loss	3255.0	3973.6	1440	480.0-5400

*The medical cost was discounted by 3%. Cost of working time loss was calculated as the hours off from work multiply the average wages per hour in 2016 in each province.

### Utilization of institutions outside hospital and the cost

Within the last year, 24.0% (671/2796), 11.8% (376/3175), and 24.6% (575/2333) visited other institutions (outside the hospital) due to myopia problems, in Anhui, Shanghai, and Yunnan, respectively.

The annual cost was listed in **Table 6**. Indirect cost, which consisted of cost of working time loss and cost of accompanying, were higher than direct cost in all the three provinces. The mean of direct cost (F = 11.67, *P* < 0.01) and cost of working time loss (F = 14.72, *P* < 0.01) were significantly different among the three provinces.

**Table 6:** Annual cost for treatment outside hospital *

Province	Variable	No.	Mean (CNY)	SD	Median (CNY)	Percentile (CNY)
**Anhui**	Direct Cost	616	238.2	423.8	50	3-300
	Cost of working time loss	664	26.3	57.7	25	0-25
	Cost of accompanying	628	119.9	367.3	50	25-100
**Shanghai**	Direct Cost	334	356.1	625.9	50	1-400
	Cost of working time loss	373	71.1	165.9	60	0-60
	Cost of accompanying	365	283.1	1386.8	60	60-180
**Yunnan**	Direct Cost	527	385.3	598.5	100	10-500
	Cost of working time loss	575	55.6	173.2	30	0-30
	Cost of accompanying	552	2576.0	54 608.9	60	30-180

*The direct cost included the cost of treatment, eye care service, and transportation, but it did not include the cost of spectacles or contact lenses. Cost of working time loss was calculated as the hours off from work multiply the average wages per hour in 2016 in each province. For students of primary school, middle school and high school, cost of working time loss was not calculated. Cost of accompanying was calculated as the hours off from work multiply the average wages per hour in 2016 in each province and the number of people accompanying.

### Prevention of myopia and the cost

In Anhui, the proportion of children who spent money for prevention of myopia in the most recent year was 14.8% (99/669), 8.0% (51/641) and 5.3% (12/225) in children aged 5-9, 10-14 and 15-19 years, respectively. In Shanghai, the proportion was 15.3% (88/576), 7.6% (31/406) and 4.0% (9/223) in children aged 5-9, 10-14 and 15-19 years, respectively. In Yunnan, the proportion is 2.8% (16/577), 5.8% (41/703) and 5.1% (17/331) in 5-9, 10-14 and 15-19 years, respectively. The average annual costs for preventing myopia were 424.6 CNY (standard deviation (SD) = 376.8 CNY), 568.2CNY (SD = 407.5 CNY) and 386.9 CNY (SD = 386.6 CNY) in Anhui, Shanghai, and Yunnan respectively. Most of the students purchased eye protection lamp and equipment to correct seating posture.

### Annual cost of treatment and prevention of myopia in urban areas of China

The cost was extrapolated for those 5 to 50 years old in the urban areas of China to explore the economic burden. It was estimated that there were 96.1 million, 31.7 million, and 15.8 million persons with myopia in the East, Middle, and West of urban China; while there were 74.7 million, 47.8 million, and 28.2 million persons without myopia in the East, Middle, and West of urban China. The national annual cost of myopia treatment and prevention classified by the middle, east and west part are shown in **Table 7**. Cost of correction accounted for the largest percentage (83.5%) in total cost of treatment and prevention. Cost of soft contact lenses composed the largest percent (49.6%) in the cost of correction, which was 41.4% of the total cost of treatment and prevention. The direct cost accounted for 90.5% of the total cost of treatment and prevention. The cost in the east part of China accounted for 77.6% of the total cost of treatment and prevention in the urban China (**Table 7**).

**Table 7:** Annual cost of myopia treatment and prevention in the urban areas of China *

		Middle	East	West	Total	Proportion (%)
**Correction**	Spectacles	2 785 861 946	10 770 780 251	2 161 653 536	15 718 295 734	
	OK or RGP	1 026 824 618	9 297 406 189	2 107 280 595	12 431 511 402	
	Soft lens	1 531 867 470	24 502 435 202	1 668 764 530	27 703 067 202	
	Total	5 344 554 034	44 570 621 643	5 937 698 661	55 852 874 338	83.5
**Hospital**	Direct	25 034 650	498 624 771	74 038 704	597 698 126	
	Indirect	500 693 009	2 719 771 480	605 771 216	3 826 235 704	
	Total	525 727 659	3 218 396 251	679 809 920	442 393 3830	6.6
**Surgery**	Direct	269 846 285	1 177 649 104	277 278 019	1 724 773 408	
	Indirect	26 198 668	395 690 099	35 475 819	457 364 587	
	Total	296 044 953	1 573 339 203	312 753 839	2 182 137 995	3.3
**Outside hospital**	Direct	348 213 800	498 172 998	385 566 131	1 231 952 929	
	Indirect	522 320 700	1 195 615 195	347 009 518	2 064 945 413	
	Total	870 534 500	1 693 788 193	732 575 649	3 296 898 342	4.9
**Prevention**		232 733 049	826 039 394	81 074 684	1 139 847 128	1.7
**Total**	Direct	6220 381 818	47 571 107 910	6 755 656 200	60 547 145 928	90.5
	Indirect	1 049 212 377	4 311 076 774	988 256 553	6 348 545 704	9.5
	Total	7 269 594 195	51 882 184 683	7 743 912 754	66 895 691 632	100.0

OK – orthokeratology, RGP – rigid gas permeable

*All presented in Chinese yuan (CNY).

Linear regression analysis showed that the total direct cost of treatment of myopia, including the cost of correction, the direct cost of treatment of myopia in medical institution and in non-medical institution, was associated with province, gender and family income (all *P* < 0.0001), but not associated with family size (*P* = 0.18). Compared with Anhui Province, the total direct cost of treatment of myopia in Shanghai and Yunnan Province increased by 270.5 CNY (95% CI = 209.0 to 332.1, *P* < 0.0001) and 257.7 CNY (95% CI = 194.5 to 320.9, *P* < 0.0001), respectively. The cost for male was 193.2 CNY (95% CI = -242.6 to -143.9, *P* < 0.0001) lower than that for female. Each year, the increase in age resulted in reduction of the annual cost by 13.7 CNY (-20.0 to -7.4, *P* < 0.0001). The higher the total family income was associated with the higher the annual cost (*P* < 0.0001) .

The total annual cost of correction and treatment (except for the cost of prevention) divided by the total myopia population in urban China, indicates that the annual cost per person is 458 CNY (69US$) for person with myopia. For non-myopic children, the annual cost per person is 35 CNY (5US$), based on the value of total cost of prevention divided by the population of non-myopia.

### Annual cost of productivity loss by myopia

**Figure 2:**
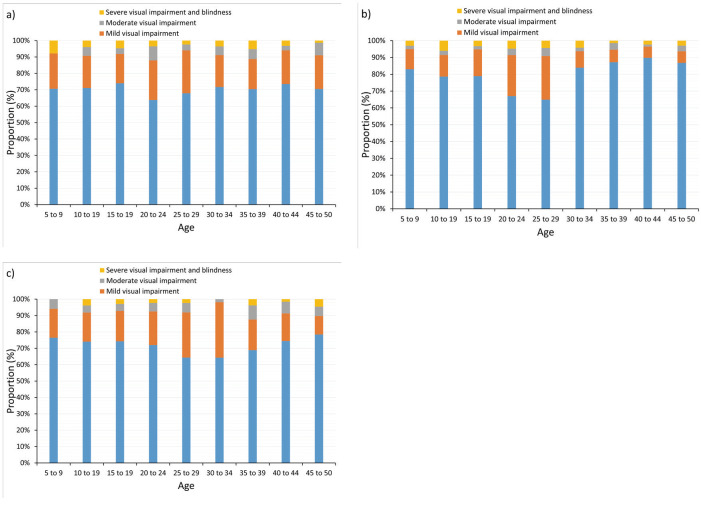
Proportion of visual impairment in Anhui, Shanghai and Yunnan. **Panel A**. Proportion of visual impairment in Anhui. **Panel B**. Proportion of visual impairment in Shanghai. **Panel C**. Proportion of visual impairment in Yunnan.

There was no statistical difference in the proportion of visual impairment between the age groups in Anhui (χ^2^ = 29.2, *P* = 0.21) and Yunnan (χ^2^ = 28.4, *P* = 0.24), while there was a statistical difference in the proportion of visual impairment between the age groups in Shanghai (χ^2^ = 132.4, *P* < 0.01). There was significant difference in the proportion of visual impairment among the three provinces (χ^2^ = 72.1, *P* < 0.01) (**Figure 2**). Combining the number of myopia patients calculated above, among urban residents aged 20 to 50 years in the Middle urban China, the number of patients with the mild to moderate visual impairment caused by myopia was 7.9 million, and the number of patients with severe visual impairment to blindness was 1.3 million. In the East, the number of patients with the mild to moderate visual impairment caused by myopia was 19.1 million, and the number of patients with severe visual impairment to blindness was 3.6 million. In the West the number of patients with the mild to moderate visual impairment caused by myopia was 3.9 million, and the number of patients with severe visual impairment to blindness was 0.4 million. Furthermore, in the population aged 20 to 50 years in urban China, the number of patients with the mild to moderate visual impairment caused by myopia was 24.8 million, and the number of patients with severe visual impairment to blindness was 4.1 million. The cost of loss of productivity was estimated to be 44.5 billion CNY for those with mild to moderate visual impairment, and 62.2 billion CNY for those with severe visual impairment to blindness.

### Total annual cost of myopia in urban China

**Figure 3:**
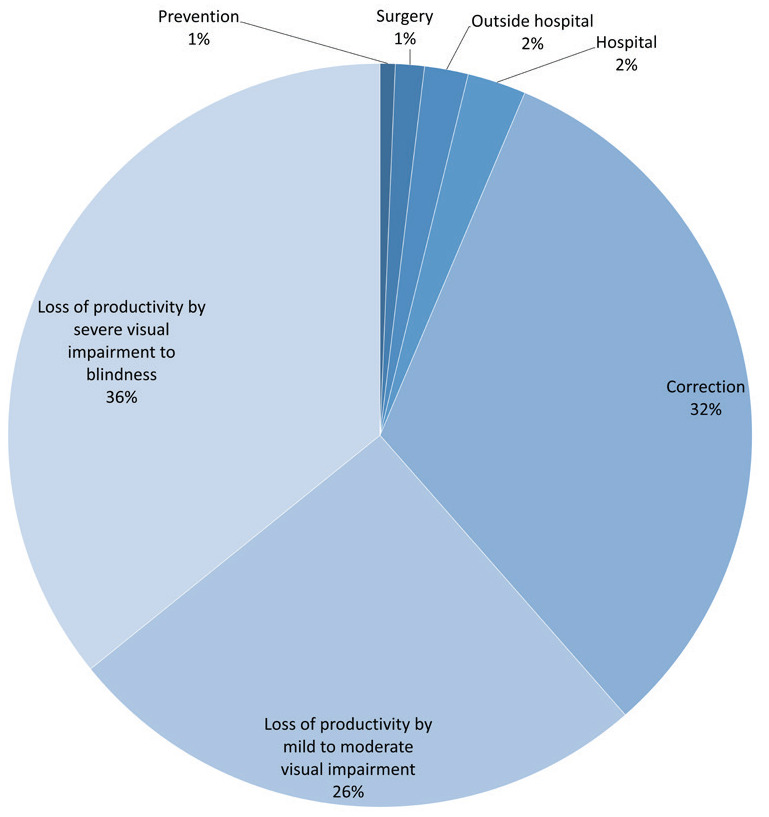
Composition of economic burden of myopia in urban China.

Combing the annual cost of treatment and prevention of myopia and the annual cost of loss of productivity, the total annual cost of myopia was 173.6 billion CNY for urban Chinese people aged 5 to 50 years, accounting for about 0.2% of the country's gross domestic product in 2016. The composition of the total annual cost of myopia was presented in **Figure 3**. The cost of productivity loss accounted for the largest percentage (61.5%) in the total annual cost, among which, the cost of productivity loss by severe visual impairment to blindness accounted for 35.8%, and the cost of productivity loss by mild to moderate visual impairment accounted for 25.6%. The cost of myopia correction accounted for 32.2% of the total cost.

### Sensitivity analyses

**Figure 4:**
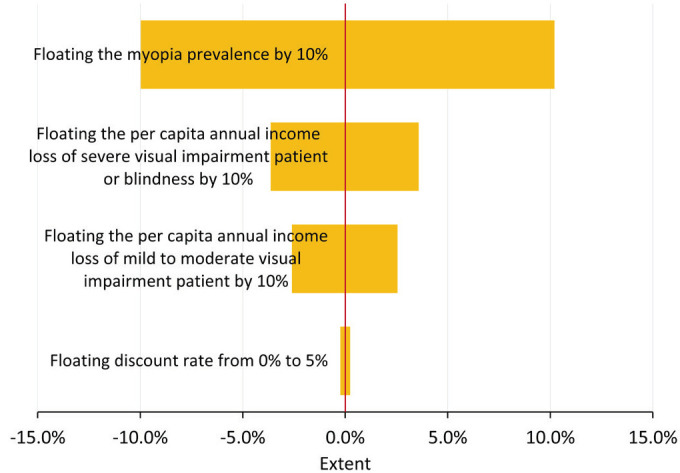
Sensitivity analyses of the economic burden of myopia in urban China.

After increasing and decreasing the prevalence of myopia by 10%, the annual cost of treatment and prevention of myopia changed from 60.4 billion CNY (9.2 billion US$) to 74.0 billion CNY (11.2 billion US$); the annual cost of loss of productivity by mild to moderate visual impairment changed from 40.0 billion CNY (6.1 billion US$) to 48.9 billion CNY (7.4 billion US$); and the annual cost of loss of productivity by severe visual impairment to blindness changed from 55.9 billion CNY (8.5 billion US$) to 68.4 billion CNY (10.4 billion US$).Floating the per capita annual income loss1 by 10% changed the annual cost of loss of productivity by mild to moderate visual impairment from 40.0 billion CNY (6.1 billion US$) to 48.9 billion CNY (7.4 billion US$). Floating the per capita annual income loss2 by 10% varied the annual cost of loss of productivity by severe visual impairment to blindness from 55.9 billion CNY (8.5 billion US$) to 68.4 billion CNY (10.4 billion US$). Altering discount rate to 0% and 5%, the cost varied from 66.5 billion CNY (10.1 billion US$) to 67.3 billion CNY (10.2 billion US$). Therefore, the sensitivity analyses suggested the cost could float from 156.3 billion CNY (23.7 billion US$) to 191.3 billion CNY (29.0 billion US$). Myopia prevalence was the most important factor affecting the economic burden (**Figure 4**).

## DISCUSSION

The extrapolated annual cost of myopia in urban China is 173.6 billion CNY (26.3 billion US$) in the 5- to 50-year-olds. The annual cost is 35 times of the annual cost of myopia in the whole Singapore (755 million US$) [10,11] and is still higher than the annual cost of correction for distance vision in all Americans aged 12 years and older (3.9-7.2 billion US$) [12]. In 2016, the annual cost of myopia in urban China accounted 0.23% of the gross domestic product [19]. Compared with other common diseases, the economic burden of myopia could rank in the top of the list in Mainland China [20-23]. Since we only calculated the cost in urban China, the cost of myopia in whole China could be much larger than the presented value, if it would include the cost of rural areas as well.

Despite the extensive total cost, the personal cost was quite trivial. The annual cost of myopia treatment and prevention per person is 458 CNY (69US$) for those with myopia and 35 CNY (5US$) for non-myopic children. In 2016, the annual income is 23821 CNY in Mainland China, and 33616 CNY in urban areas [19]. The cost per capita in urban China is relatively low compared with that of Singapore (83US$ for myopic children and adolescents, and 709US$ for adults older than 40 years) [10,11]. In the United States, the median cost of a pair of spectacles was 92 to 180US$ [12]. Based on the present data, the estimated population with myopia is 31.7 million, 96.1 million and 15.8 million in the urban areas of middle, east and west part of China, respectively. Therefore, the higher annual cost in the urban China could resulted from the large size of population with myopia, which also explained the large percentage (77.6%) of the total cost in east urban China.

In urban areas, for myopic person, the correction rate was over 85%, which was relatively high compared with that in rural areas [15,24]. However, utilization of medical service is not relatively low. About 23.8%, 16.5%, and 37.4% of myopic children went to visit hospitals in the past one year of the study period in Anhui, Shanghai and Yunnan. Use of OK lenses and RGP lenses were also small proportions, ranging from 0%-9.1% in people younger than 20 years. Wearing orthokeratology lenses could retard the progression of myopia compared with wearing spectacles [25], hence preventing future incidence of high myopia. The data indicated that most myopia have only corrected the refractive error without go to doctors for treatment to retard the progression of myopia. The medical services have not been thoroughly used to control the myopia progression, which could result in the increasing prevalence of high myopia [26].

In the present study, the most economical way to correct myopia could be using spectacles. The annual cost per person was cheaper compared with using orthokeratology or RGP lenses and using soft contact lenses. In European countries, economic evaluation has shown that compared with contact lenses, myopia surgery is cost-effective technology to correct myopia in a long run [7,8]. In the present study, the mean cost of myopia surgery ranged from 10759 CNY to 16570 CNY, the mean cost of using spectacles ranged from 155 CNY to 314 CNY, and the mean cost of wearing soft contact lenses ranged from 951 CNY to 1740 CNY. Using the median value to calculate, the cost of myopia surgery equals to about 10 years of using soft contact lenses and 58 years of using spectacles.

In addition to calculating the cost of treatment and prevention of myopia, our study also calculated the cost of loss of productivity caused by the impaired vision associated with myopia, and confirmed that the cost incurred by the latter was much more serious than the former. It was estimated that the global potential productivity loss associated with the burden of myopia related visual impairment in 2015 was about 250 billion US$ using review, meta analyses, and modeling, however, the study didn’t calculate the direct cost by myopia[4]. The present study estimated that the annual cost of loss of productivity was 16.1 billion US$, accounting for the 61.5% of the total annual cost. Although using different calculation methods and the different investigation population, the results of our study cannot be directly compared with the previous study [4], the present study verified the hypothesis that the cost by loss of productivity could be substantially larger than the cost of treatment and prevention of myopia. Therefore, the focus of myopia prevention and control should be the prevention of visual impairment caused by myopia, including uncorrected refractive error, and irreversible visual impairment caused by high myopia. How to prevent myopia, how to improve the myopia correction rate and how to control the development of myopia into high myopia should be the key issues to decrease the myopia disease burden.

This is the first nationwide study to calculate the burden of myopia in mainland China. The study collected both direct and indirect costs, and compared their compositions. Meanwhile, it is also the first investigation on the utilization of myopia medical services. The results provided an important basis for the formulation of China's myopia prevention and control policy, and point out the main focus of myopia prevention and control. In addition, the results also have certain reference value for the prevention and control of global myopia, especially for East Asia or other countries with similar level of economic development as China. Areas with low prevalence of myopia should also beware of the increase in the prevalence of myopia and control its prevalence in time, because the disease burden of myopia to both society and individuals is tremendous.

There are some limitations in the present study. First, the study population was younger than 50 years, which is younger than the common age of onset of high myopia related complications [27], and thus the related costs were not calculated. The complications caused by high myopia usually lead to severe visual impairment and even blindness, and potentially large treatment cost, such as cost for anti-VEGF injections, vitrectomy surgery and low vision rehabilitation services [28,29]. The global annual cost of productivity loss due to myopic macular degeneration, one of the visually impaired complications of high myopia, was estimated to be 6 billion US$ [4]. Therefore, the present study underestimated the economic burden of myopia if calculated in the general population. Second, the self-reported questionnaire cannot avoid false declaration of utilization and cost, despite that quality control was done while filling the questionnaires and was checked again upon the completion of the questionnaires. Also. the self-reported myopia will cause some errors in the estimation of the prevalence of myopia and visual impairment, since the gold standard for diagnosing myopia is cycloplegic refraction [30]. Our study has possibly underestimated the prevalence of myopia, especially for children [31,32], since the awareness rate of myopia was only about 60% in Chinese parents [33]. However, the lower self-reported myopia prevalence may not have substantial influence on the calculation of cost. Since if the parents don’t know their children have myopia, cost associated with myopia correction and treatment may not incur. In the sensitivity analyses, we increased the prevalence by 10%, and the total cost increased by 10% as well. With the increasing prevalence of myopia, the disease burden of myopia could become heavier and heavier. Third, for the population older than 24 years, the study included the students’ parents, which could cause bias in the results. However, there is no evidence that family with children could differ in the use of health service from those without children. In addition, because of the availability of data, we only collected the cost of myopia for the urban population aged 5-50 years, without collecting the rural population. This part of the population may cause more economic losses due to uncorrected refractive errors. Last but not the least, despite that the random cluster sampling method was used to obtain representative sample, and the national census data was used to correct the diverse population structure in different parts of China, potential bias may also exist when extrapolating the results to the whole urban China.

## CONCLUSIONS

The present study presented a large economic burden of myopia in urban China. The major reason for the burden is the large population of myopia person. The main composition of the cost came from the cost of productivity loss due to myopia, and the cheapest way to correct myopia is using spectacles. Therefore, strategies are in urgent need to control the prevalence of myopia and visual impairment caused by myopia. Children who are already myopic should be encouraged to accept formal medical diagnosis and treatment, such as atropine drops and orthokeratology lenses, to retard progressing into high myopia. Government needs to consider strategies to rationally allocate the medical resources to decrease the myopia related burden.
